# Methodological challenges in assessing the impact of comorbidities on costs in Alzheimer’s disease clinical trials

**DOI:** 10.1007/s10198-014-0648-7

**Published:** 2014-11-20

**Authors:** Kristin Kahle-Wrobleski, Howard Fillit, Jonathan Kurlander, Catherine Reed, Mark Belger

**Affiliations:** 1 Eli Lilly and Company, Lilly Corporate Center, Indianapolis, IN 46285 USA; 2 Alzheimer’s Drug Discovery Foundation and Icahn School of Medicine at Mount Sinai, New York, NY USA; 3 Optum, Eden Prairie, MN USA; 4 Eli Lilly and Company Limited, Windlesham, UK

**Keywords:** Alzheimer’s disease, Randomized controlled trials, Semagacestat, Comorbidities, Concomitant medications, Economics, I120 (health production), C900 (design of experiments)

## Abstract

**Background:**

Alzheimer’s disease (AD) is associated with considerable costs and has a significant impact on health and social care systems.

**Objective:**

This study assessed whether baseline comorbidities present in 2,594 patients with AD participating in two semagacestat randomized placebo-controlled trials (RCTs) would significantly impact overall costs.

**Methods:**

Resource utilization was captured using the Resource Utilization in Dementia Scale-Lite. Comorbidities and concomitant medications were tabulated via patient and caregiver reports. Only baseline data were analyzed. Direct and indirect costs per month were calculated per patient. The relationship between cost and explanatory variables was explored in a regression model.

**Results:**

The baseline monthly cost of care in this RCT population was £1,147 ± 2,483, with informal care costs accounting for 75 % of costs. Gender, age, and functional status were significant predictors of costs (*p* ≤ 0.0001). The cost ratio was not impacted when the number of comorbidities was added to the model (cost ratio = 0.95; 95 % CI 0.91–0.99) or when combined with the number of concomitant medications (cost ratio = 0.97; 95 % CI 0.95–1.00). Inconsistent findings related to the impact of individual comorbidities on costs were noted in sensitivity analyses.

**Conclusions:**

The number of comorbidities, alone or when combined with concomitant medications, did not impact baseline costs of care, perhaps because RCTs often enroll less severely ill and more medically stable patients. However, higher costs were consistently associated with greater functional impairment similar to non-RCT databases. Supplemental sources (e.g., claims databases) are likely needed to better estimate the effects of disease and treatment on costs of illness captured in RCTs for AD.

## Introduction

Alzheimer’s disease (AD) is progressive neurological disorder that is thought to be the causal pathology in roughly 70 % of dementia cases [1]. AD is a considerable and growing public health issue with a significant impact on health and social care systems [1, 2]. The estimated costs of caring for patients with dementia worldwide in 2010 reached $604 billion [3], and those figures are expected to continue rising as the world population ages [3, 4].

Patients with AD incur far greater costs (inpatient and outpatient care and prescriptions) than their cohorts without AD. For patients with AD, inpatient stays and institutionalization account for the majority of direct care costs [5, 6]. Additionally, the informal care costs (e.g., number of annual unpaid care hours per unprofessional caregiver) of patients living in the community represent a substantial proportion of overall costs of care [5, 7, 8].

Costs are influenced by a variety of factors, including severity of the illness [7, 9] and patient comorbidities [5, 9–13]. Patients with dementia or AD typically have more comorbidities than matched controls [11, 13]. The most common comorbidities include psychiatric (specifically depression), diabetes and other endocrine/metabolic disorders, cerebrovascular and cardiovascular disease, gastrointestinal disorders, urogenital disorders, and hip fractures [12–15]. These comorbidities, either alone or through interactive effects with dementia/AD, in addition to the prescription cost for treatment of non-AD illnesses, contribute to the overall greater cost associated with AD. Furthermore, it has been demonstrated that the care costs of patients with AD increase with greater dementia severity [5, 8, 12, 13].

Considerable efforts are underway to find new treatments that will modify the underlying pathological process of AD [1]. While regulatory evaluations of new AD treatments will focus on safety and efficacy [2, 4], economic data and cost-effectiveness modeling techniques are needed to inform payer evaluations.

Given the difficulties in capturing costs of care in parallel with clinical measures over a substantial period of time to test disease-modifying agents, previous cost-effectiveness analyses of AD therapies have relied on multiple data sources [16–18]. Cost-of-illness and disease progression data from sources external to the randomized controlled trials (RCTs) have been used in combination with efficacy findings from RCTs [18]. These cost sources included observational studies as well as administrative claims databases [3]. Ideally, economic data would be incorporated into clinical trials, particularly as more recent trials of disease-modifying treatments for AD span 18 months. For instance, Gustavsson et al. [19] used placebo data from a negative, 18-month trial of an ostensible AD disease modifier to examine predictors of care costs, excluding comorbidities and concomitant medications. Their findings demonstrated similar patterns to what is commonly seen in non-RCT studies, which is that a greater degree of functional, cognitive, and behavioral impairment was significantly associated with higher and increasing costs over time [19]. Care costs also increased over time. A better understanding of the comorbid conditions in the RCT population and their potential impact on care costs may provide greater insight into the effectiveness of treatments for chronic diseases like AD and more accurate estimations of the costs associated with the overall disease state and treatment [20].

The present analyses used baseline data from a discontinued clinical trial to examine how comorbidities would impact costs of illness, as assessed in the trial. It was hypothesized that that the comorbidity profile would have a significant impact on overall costs, similar to patterns seen in observational and retrospective claims analyses. If the hypothesis is valid, evaluating the type of comorbidities at baseline and over time in clinical trials may be an important added covariate in assessing the overall impact of future pharmacotherapeutic candidates on costs.

## Methods

The present analyses used baseline data from two similarly designed 88-week RCTs of semagacestat [H6L-MC-LFAN (IDENTITY) and H6L-MC-LFBC (IDENTITY-2)], a gamma-secretase inhibitor AD drug candidate previously in development, in patients with mild-to-moderate dementia due to AD [21]. Further methodological details for the H6L-MC-LFAN and H6L-MC-LFBC trials were previously reported [21] and are also provided at http://www.clinicaltrials.gov/ct2/show/NCT00594568?term=LFAN&rank=2 and http://www.clinicaltrials.gov/ct2/show/NCT00762411?term=LFAN&rank=3, respectively. The experimental treatment was discontinued early due to safety findings. The protocols were subsequently amended prior to completion in order to allow for continued safety monitoring of patients [21]. As a result of the early discontinuation of study drug and subsequent protocol amendments necessitating the change of the study design, the current analysis in this paper relied on baseline data only (with data from both the treatment and placebo arms included). Between the two studies, clinical trials were conducted in 31 countries. Randomized patients met criteria for probable AD according to the National Institute of Neurological and Communicative Disease and Stroke/Alzheimer’s Disease and Related Disorders Association (NINCDS-ADRDA) guidelines and were in the mild-to-moderate stage of dementia severity as measured by the Mini-Mental State Examination (MMSE 16–26, inclusive). Assessment of inclusion/exclusion criteria was based on patient age (≥55 years), patient and caregiver reports, investigator assessment, and medical records if available. Relevant to the present analyses, patients were excluded if they suffered from life-threatening or serious chronic illnesses, or unstable illness (e.g., significant active cardiac disease, uncontrolled hypertension, uncompensated congestive heart failure, endocarditis, or end-stage renal disease) which, in the opinion of the investigator, would interfere with assessment of safety and efficacy or that would result in a life expectancy of less than 2 years. Patients were required to have a reliable caregiver familiar with their health and functional status. Informed consent was obtained from all participants or their legal representatives prior to study enrollment. The studies were conducted according to the Declaration of Helsinki and were approved by local independent review boards in all countries.

Co-primary efficacy measures for the trial were the 11-item Alzheimer’s Disease Assessment Scale-Cognitive Subscale (ADAS-Cog) [22, 23] and the Alzheimer’s Disease Cooperative Study-Activities of Daily Living Scale (ADCS-ADL) [24, 25] for functional ability. Additional outcome measures included in the trials were the Mini-Mental State Examination (MMSE) [26], Clinical Dementia Rating (CDR) scale [27, 28], Neuropsychiatric Inventory (NPI) [29], and EuroQol 5-Dimension (EQ-5D) [21, 30].

Resource utilization was assessed by the Resource Utilization in Dementia-Lite (RUD-Lite) scale [31, 32]. This scale includes a structured interview completed with the AD patient and their caregiver to assess healthcare resource utilization of patients and their caregivers. In these studies, caregivers provided information on the RUD-Lite regarding the number of hours (in the past month, at baseline) that care was provided to help the patient with basic (e.g., eating, dressing, etc.) and instrumental activities of daily living (ADLs; e.g., shopping, cooking, etc.), as well as the number of hours spent supervising the patient. Additional resource utilization measures assessed included the number of hospital visits, living accommodations (e.g., nursing home placement), and use of community resources.

Patient comorbidities and concomitant medications at baseline were assessed primarily via patient and caregiver reports at study entry as well as via medical records, when available. Individual drugs were grouped according to drug class to facilitate reporting. Specific comorbidities were chosen for inclusion in the subsequent analyses that represented common age-related comorbidities in the study population most likely to be afflicted with AD; concomitant medications were selected based on the most frequently identified comorbid conditions.

## Analyses

Only baseline data were analyzed and are presented. Summary statistics for the clinical scales and the profile of comorbidities and number of concomitant medications were generated. Although study inclusion criteria defined mild-to-moderate dementia severity by an MMSE score ranging from 16 to 26, the scores at baseline reflected a much broader range. Accordingly, dementia severity in the analyses herein was categorized as mild (20–30), moderate (10–19), and severe (0–9) using the standard convention. Mean resource utilization captured by the RUD-Lite, categorized by type of resource, was tabulated. Baseline report of caregiver time spent on personal ADLs, instrumental ADLs, and supervision in the past month is presented as mean hours per month and aggregated into a mean of total informal care time spent per month. The total caregiver time was capped at 540 h per month (18 h/day) to avoid overestimation. Each type of accommodation and hospitalization is presented as mean number of days per month, and community care is presented as hours per month.

Direct and indirect costs per month were calculated for each patient in the study. All cost calculations were based multiplying RUD-Lite resource utilization unit estimates by published reimbursement costs in the United Kingdom (UK) (Table 1) to allow for a common metric across countries and to allow for comparison to recent publications on cost of illness [8]. A sensitivity analysis of the unit costs from the United States (US) was performed to confirm if the factors were associated with the resource use or the cost of those services. The model results for the US were generally consistent with UK costs (results not shown). The direct costs were based on the use of accommodation (intermediate housing, dementia-specific residential housing, long-term institutionalization), hospitalizations (geriatric, psychiatric, internal medicine, and surgery), and community care visits (district nurse, home-support worker, home-delivered meals, transportation, and day care). Indirect costs were based on cost of caregiver lost production calculated as the unadjusted sum of instrumental and personal activities of daily living with a maximum of 540 h (employed cut-off of 65 years of age) and at the value of leisure time (retired/not employed). Missing data were excluded from all statistics (e.g., sample size, percentage, mean, standard deviation) in all tables. Cost analyses were based on the whole patient population. For regressions, if any of the covariates were missing, the entire observation was ignored.

**Table 1:** Unit costs (UK British Pound Sterling)

Resource	Unit cost (£, 2011)
Informal care (per hour)	
Lost production^a^	12.03
Lost leisure time^b^	4.21
Accommodation (per night)^c^	
Intermediate forms of accommodation	60.00
Dementia-specific residential accommodation	71.00
Long-term institutional care (nursing home)	103.00
Hospitalization (per night)^c^	
Geriatrics	319.00
Psychiatric	319.00
Internal medicine	319.00
Surgery	319.00
Community care services (per/hour/session/item)^c^	
District nurse home visit, per hour	73.00
Home support worker/home care, per hour	29.00
Day care, per session	36.00
Home-delivered meals, per meal	5.00
Transportation, per ride^d^	8.15^e^

^a^Estimated at the mean hourly gross pay for full-time employees in the UK [33] plus an 8 % average employer pension contribution according to FSTE 100 [34]

^b^Lost leisure time based on 35 % of gross average wage rate [35]

^c^Cost of hospitalization (per night) based on hospital ward of service [36]

^d^Rates of taxi and private hire vehicle [37]

^e^Inflated from 1999 year price levels to 2011 year price levels by overall consumer price index [37, 38]

Differences in mean costs between patient subgroups were tested for statistical significance using unpaired *t* tests.

A cluster analysis using the Ward’s method of minimum variance to cluster countries with similar baseline healthcare costs was performed to examine potential differences in cost structures between countries. Due to the high number of countries missing at least one cost component, countries were clustered based on total monthly costs per patient. Results from an RCT do not need a cluster adjustment. However, the potential effect due to the clustering of patients within a site was considered. This, in combination with the small number of patients within each site, suggested that there was no need for a clustering adjustment.

The relationship between cost of care and explanatory variables was explored in a regression model to determine the drivers of cost of care. Cost and resource data are usually of a distribution that constitutes of large number of zero observations, no negative observations, and a few very high observations. Cost functions are therefore estimated with a generalized linear model (GLM) [30]. The GLM gamma model with a log link was fitted using the maximum likelihood approach and was used to estimate the cost function. A sensitivity analysis using an ordinary least-squares approach yielded similar results (results not shown).

Comorbidities, the number of comorbidities, and the number of concomitant medications, including AD medications, were the covariates to impact costs. Adjustment variables added to the costs model included demographics of the patient (age, gender), cognitive (MMSE), functional (ADCS-ADL), behavioral (NPI), and global (CDR, CDR-SB) disease indicators. The comorbidities selected as potential predictors included cancer, chronic obstructive pulmonary disease, depression, diabetes, hyperlipidemia, hypertension, cardiac ischemia, urinary tract infection, epilepsy, and stroke. Individual comorbidities were selected to represent common age-related comorbidities and to facilitate comparison to other samples (e.g., ICTUS observational study [14]).

## Results

A total of 2,594 patients were included at baseline, 55 % were female with a mean age of 73 ± 8 years. As shown in Table 2, at baseline, 61 % of patients had mild dementia (MMSE 20–30), 32 % were in the least impaired quartile of ADL ability (ADCS-ADL 68–78), 64 % had a low number of neuropsychiatric symptoms (NPI < 10), and approximately 87 % were rated as mild on a global performance scale (CDR 0.5–1). Of note, although the protocol inclusion requirement restricted enrollment to patients with mild-to-moderate dementia at the screening visit, one patient did score in the severe dementia range at the baseline study visit and was included in all analyses. Of the comorbidities included in the analyses, hypertension and hyperlipidemia were most commonly observed. Nearly all patients (94 %) in this RCT population were taking at least one medication inclusive of those for AD (Table 3).

**Table 2:** Baseline demographics and by instruments

Demographics	Total (*N* = 2,594)	
Male, *n*	1,161 (44.76 %)	
Age, mean (SD)^a^	73.26 (7.86)	
Disease severity measure	*n*	%
MMSE, mean score (SD)	20.68 (3.45)	
Mild (20–30)	1,594	61.47
Moderate (10–19)	998	38.49
Severe (0–9)	1	0.04
ADCS-ADL, mean score (SD)	58.48 (13.96)	
68–78 (least dependent)	816	31.57
58–67	743	28.74
46–57	556	21.51
0–45 (most dependent)	470	18.18
NPI, mean score (SD)	9.59 (10.72)	
High (≥10)	942	36.48
Low (<10)	1,640	63.52
CDR, mean score (SD)	0.95 (0.49)	
Mild (0.5–1)	2,236	86.70
Moderate (2)	333	12.91
Severe (3)	10	0.39
CDR-SB, mean score (SD)	5.61 (2.84)	
Mild (0.5–9)	2,284	88.46
Moderate (9.5–15.5)	295	11.43
Severe (16–18)	3	0.12

*ADCS-ADL* Alzheimer’s Disease Cooperative Study-Activities of Daily Living, *CDR* Clinical Dementia Rating, *CDR-SB* Clinical Dementia Rating–Sum of Boxes, dimensions, *MMSE* Mini-Mental State Examination, *N* overall sample size, *n* number of patients in category, *NPI* Neuropsychiatric Inventory, *SD* standard deviation

^a^Age breakdown in years includes: 55–64 (*n* = 444), 65–74 (*n* = 961), and ≥75 (*n* = 1,188)

**Table 3:** Baseline comorbidity and medications

Comorbidity^a^		Medications^b^		Number of medications^c^	
Total (*N* = 2,594)					
	*n* (%)		*n* (%)		*n* (%)
Cancer	24 (0.92)	Alzheimer’s disease treatments		None	146 (5.63)
COPD	48 (1.84)	Donepezil	1,338 (51.58)	1–2	1,006 (38.78)
Depression	642 (24.66)	Galantamine	346 (13.34)	3–4	885 (34.12)
Diabetes	294 (11.29)	Rivastigmine	383 (14.76)	5+	557 (21.47)
Hyperlipidemia	968 (37.19)	Memantine	787 (30.34)		
Hypertension	1,097 (42.14)	Other concomitant medications			
Cardiac ischemia	259 (9.95)	ACE inhibitors	494 (19.04)		
Urinary tract infection	24 (0.92)	Angiotensin II antagonists	302 (11.64)		
Epilepsy	13 (0.50)	Antidepressants	756 (29.14)		
Stroke	30 (1.15)	Beta-blocking agents	435 (16.77)		
		Biguanides	139 (5.36)		
		Corticosteroids	132 (5.09)		
		Dihydropyridine derivatives	275 (10.60)		
		Diuretics, thiazides, and potassium-sparing agents	178 (6.86)		
		HMG CoA reductase inhibitors	880 (33.92)		
		Sedatives	370 (14.26)		
		Thyroid hormones	283 (10.91)		
		Vitamin B12	191 (7.36)		

*ACE* angiotensin converting enzyme, *COPD* chronic obstructive pulmonary disease, *HMG CoA* hydroxymethylglutaryl coenzyme A, *N* overall sample size, *n* number of patients in category

^a^Comorbid conditions occurring in <2 % included: blood and lymphatic system disorders, ear, endocrine, eye, general disorders and administration site conditions, hepatobiliary, immune system disorders, investigations, metabolism, musculoskeletal and connective tissue, neoplasm, renal, reproductive system and breast disorders, respiratory, and skin

^b^Medication use occurring in <2 % of patients included: antiarrhythmics, butyrophenone derivatives, digitalis glycosides, and insulins

^**c**^Inclusive of medications for Alzheimer’s disease

The baseline monthly cost of care in this RCT population was £1,147 ± 2,483 for which informal care costs accounted for 75 % of costs (Table 4). Total costs per patient per month stratified by gender and age were numerically greater for females than males (£1,247 vs. £1,023) and numerically greater for patients 75 years and older (£1,352 for 75 + years, £832 for 65–74 years, and £1,281 for 55–64 years).

**Table 4:** Baseline utilization and cost of care

Resource item	Baseline (*N* = 2,594)				
	Resource utilization			Cost	
	*n* ^a^	Mean^a^	SD	Mean (UK £^b^)	SD
Informal care	2,578	173.47 h/month	243.01	855.19	1,266.62
Informal care (lost production)^c^	2,578	104.31 h/month	126.35	1,254.82	1,520.02
Accommodation	29	0.06 day/month	1.04	3.81	63.74
Intermediate forms of accommodation	24	0.05	1.03	3.12	61.56
Dementia-specific residential accommodation	6	0	0.04	0.05	2.79
Long-term institutional care (nursing home)	9	0.01	0.16	0.64	16.42
Hospitalization	110	0.05 day/month	0.92	15.99	293.4
Geriatrics	100	0	0.08	0.49	25.05
Psychiatric	103	0.03	0.82	10.94	261.01
Internal medicine	101	0.01	0.41	3.69	129.85
Surgery	100	0	0.08	0.86	24.25
Community care services	2,244	8.61 h/month	52.38	272	1,912.51
District nurse	2,106	0.42	14.29	30.31	1,043.16
Home support worker	2,108	3.3	31.74	95.57	920.38
Home-delivered meals	2,076	0.48	9.31	2.41	46.54
Transportation	2,100	0.55	6.75	4.46	55.03
Total cost of care^d^	–	–	–	1,146.98	2,483.02
Total cost of care with informal care as all lost production^e^	–	–	–	1,546.62	2,631.60

*N* overall sample size, *n* number of patients in category

^a^ *n* patients or caregivers reporting any use; mean includes all patients (users and nonusers) with missing values set to zero

^b^Costs are per subject per month (in UK pounds)

^c^Lost production is the unadjusted sum of instrumental and personal activities of daily living (PADL) (with a maximum of 540) regardless of caregiver age 65+

^d^Total cost of care includes informal care costs using lost leisure time for non-working caregivers (£855.19)

^e^Total cost of care includes informal care costs using all lost production for non-working caregivers (£1,254.82)

The individual effects of patient demographics and disease severity on total costs of care were estimated in a cost model using gamma model distribution with a log link. Gender, age, and functional status were all significant predictors of costs (*p* ≤ 0.01). In this RCT population, age was a significant variable associated with baseline total costs of care. Patients <65 years old and older patients (>75 years) had significantly (*p* < 0.0001) greater costs compared to patients aged 65–75 (Table 5). When individual comorbidities were added to the model, cancer was significantly associated with higher baseline costs (*p* = 0.003), whereas cardiac ischemia is significantly associated with lower total costs (*p* = 0.002; Table 6). The cost ratio was not impacted when the number of comorbidities (cost ratio = 0.95; 95 % CI 0.91–0.99) was added to the model (as proxies of comorbid severity/medical complexity) or when combined with the number of concomitant meds (cost ratio = 0.97; 95 % CI 0.95–1.00), despite reaching statistical significance (*p* ≤ 0.03). In sensitivity analyses using US unit costs, the association between cancer and higher costs disappeared, but cardiac ischemia remained as a predictor of lower costs and hyperlipidemia was also associated with a lower cost ratio. The impact of gender, age, and functional status remained significant.

**Table 5:** Baseline demographics and clinical predictors for total cost of care (UK costs)—gamma model with missing costs

Independent variables	Estimate	Standard error	Cost ratio			
			Ratio	Lower 95 % CI	Upper 95 % CI	*p* value
Gender: male (ref: female)	−0.1266	0.0485	0.88	0.80	0.97	**0.009**
Age: 65–75 (ref: 75+)	−0.2334	0.0533	0.79	0.71	0.88	**<0.0001**
Age: 55-64 (ref: 75+)	0.2951	0.0684	1.34	1.18	1.54	**<0.0001**
MMSE: mild (20–30) (ref: 10-19)	0.1015	0.0543	1.11	1.0	1.23	0.062
ADCSADL: 68–78 (ref: 0-45)	−0.4390	0.0862	0.64	0.55	0.76	**<0.0001**
ADCSADL: 58–67 (ref: 0-45)	−0.6217	0.0829	0.54	0.46	0.63	**<0.0001**
ADCSADL: 46–57 (ref: 0–45)	−0.3481	0.0818	0.71	0.60	0.83	**<0.0001**
NPI: <10 (ref: 10+)	−0.0723	0.0523	0.93	0.84	1.03	0.167
CDR: 0.5–1 (ref: 2+)	−0.0064	0.4032	0.99	0.45	2.19	0.987
CDR: 2 (ref:3)	0.0983	0.3876	1.10	0.52	2.36	0.8
CDR-SB: 0.5–9 (ref: 16–18)	0.2876	0.5082	1.33	0.49	3.61	0.571
CDR-SB: 9.5–15.5 (ref: 16–18)	0.4562	0.4952	1.58	0.60	4.17	0.357

*p* < 0.01 shown as* bolded text*

*ADCS-ADL* Alzheimer’s disease cooperative study: activities of daily living, *CDR* Clinical Dementia Rating, *CDR-SB* Clinical Dementia Rating-Sum of Boxes, *CI* confidence interval, *MMSE* Mini-Mental State Examination, *NPI* Neuropsychiatric Inventory, *ref* reference

**Table 6:** Baseline comorbidities as predictors of total cost of care (UK costs)—gamma model with missing costs and comorbidities

Independent variables	Estimate	Standard error	Cost ratio			
			Ratio	Lower 95 % CI	Upper 95 % CI	*p* value
Gender: male (ref: female)	−0.1365	0.0486	0.87	0.79	0.96	**0.005**
Age: 65–75 (ref: 75+)	−0.2374	0.0536	0.79	0.71	0.88	**<0.0001**
Age: 55–64 (ref: 75+)	0.2809	0.0696	1.32	1.16	1.52	**<0.0001**
ADCS-ADL: 68–78 (ref: 0–45)	−0.5174	0.0709	0.60	0.52	0.68	**<0.0001**
ADCS-ADL: 58–67 (ref: 0–45)	−0.7223	0.0714	0.49	0.42	0.56	**<0.0001**
ADCS-ADL: 46–57 (ref: 0–45)	−0.4364	0.0762	0.65	0.56	0.75	**<0.0001**
Cancer	0.7595	0.2580	2.14	1.29	3.54	**0.003**
COPD	−0.1016	0.1770	0.90	0.64	1.28	0.566
Depression	0.0257	0.0566	1.03	0.92	1.15	0.65
Diabetes	−0.0387	0.0763	0.96	0.83	1.12	0.612
Hyperlipidemia	−0.1144	0.0518	0.89	0.81	0.99	0.027
Hypertension	−0.0187	0.0495	0.98	0.89	1.08	0.706
Cardiac ischemia	−0.2554	0.0806	0.77	0.66	0.91	**0.002**
UTI	−0.2902	0.2489	0.75	0.46	1.22	0.244
Epilepsy	0.1166	0.3351	1.12	0.58	2.17	0.728
Stroke	0.5269	0.2220	1.69	1.10	2.62	0.018

Significant *p* values are shown as* bolded text*. Model retained significant demographic and clinical predictors of care costs

*ADCS-ADL* Alzheimer’s disease cooperative study: activities of daily living, *CI* confidence interval, *COPD* chronic obstructive pulmonary disease, *ref* reference, *UTI* urinary tract infection

## Discussion

The present analysis looked at the impact of comorbid conditions in addition to demographics, disease severity, and number of concomitant medications on baseline costs in a clinical trial population of patients with mild-to-moderate AD. The number of comorbidities did not yield an impact on baseline total costs of care, and combining the number of comorbid conditions and concomitant medications to represent comorbid severity had a very nominal impact on baseline costs. In this analysis, higher costs were associated with greater functional impairment and are consistent with similar published analyses [19, 39–41]. Regardless of this unexpected disassociation between total number of comorbidities and costs, it is important to recognize that results were dependent on the clinical trial population studied. Accordingly, there is a benefit in understanding how findings vary across relevant data generated from claims analyses, RCTs, and observational studies.

Comorbidity had a minimal impact on cost, whether individually or expressed as severity (number of conditions). A more consistent impact of comorbidities on higher costs of care was hypothesized. Unexpectedly, cardiac ischemia was a predictor of lower costs, whereas in claims-based samples, patients with a history of cardiac disease had higher costs due to more frequent hospitalizations, preventable hospitalizations, etc. [13]. This suggests that patients with a history of cardiac ischemia who enroll in clinical trials potentially represent a subset of patients with cardiac ischemia who are managed more appropriately and also who are more responsive to treatments for their comorbidities. Additionally, clinical trials are not powered specifically for economic endpoints or analyses. Due to a restricted range of comorbidity severity in clinical trials, the lack of findings may also reflect a general trend of more medically stable and less medically ill patients with AD enrolling in clinical trials. This is likely to create a disconnect in findings between claims databases, observational studies, and RCTs in that RCTs are unlikely to include the heterogeneity of patients found in non-randomized studies. Finally, while the type of comorbidity impacting cost also varied slightly depending whether UK or US unit costs were used, this difference did not change the fact that comorbidity in these analyses was unassociated with escalated costs.

Baseline rates of comorbid conditions were lower than those described in claims database studies [13] but consistent with prospective observational studies [11, 14, 42]. Clinical trial populations are generally expected to have fewer comorbid conditions given the study inclusion/exclusions criteria for allowable comorbidities and severity of comorbidities [20]. Baseline costs for RCT populations may therefore be different from populations in claims databases or community-based studies in that the population sample is not likely to reflect a true distribution of the severity of comorbid diseases. Furthermore, participation in 18-month AD clinical trials is, in part, contingent upon the caregiver’s willingness and motivation [7]. Therefore, these patients may be more clinically stable or better managed, and may represent a subpopulation without significant other comorbidities who responded well to therapeutic intervention. It is important to note that, at the time of these clinical trials, diagnosing a patient as having probable AD required a rigorous screening of the influence of any comorbidities on cognitive/functional impairment consistent with the NINCDS-ADRDA criteria. A selection bias may have been inadvertently created for patients well enough to participate or more motivated to participate in trials, or who had not had any significant exacerbations of medical conditions that would prevent inclusion in a clinical trial.

A challenge in self-reported methods for comorbidities in clinical trials is that it does not allow for an easy comparison with baseline rates of comorbid disorders within the healthcare system of interest. If access to actual medical records was included as part of trial consent, this would provide an opportunity to characterize the similarities and differences of a matched cohort within the same healthcare system, as well as help to understand baseline rates of diseases and acute events. Access to claims data may also eliminate some issues with proxy reporting of resource utilization, such as unawareness of care use or a too-long recall period [7, 8]. In addition, pre- and post-trial trajectories of resources use would be available for analysis. Claims data has its limitations, however, and important variables, such as use of community services and caregiver hours, would still need to be assessed via self/caregiver reports [43].

Future studies should consider evaluating the impact of comorbid conditions longitudinally within clinical trial populations. As demonstrated in claims databases or community-based studies, costs associated with comorbid conditions increase over time [9, 12]. Collection of costs longitudinally at periodic intervals may inform the potential indicators for some stability of costs over time. Unfortunately, due to the early discontinuation of treatment dosing within this trial and subsequent protocol amendment, the reliability and validity of follow-up cost data were not considered to be of significant enough rigor to allow for a robust study of longitudinal costs. The available longitudinal data included variable lengths of follow-up and forcibly smaller sample sizes at later time points that would not reflect typical study attrition.

Although conclusive statements about the impact of comorbidities on costs could not be made with the present cross-sectional analyses, it is important to note that consistencies with the previous literature were noted, particularly around the association between greater functional impairment and costs [44]. It is well appreciated in geriatric medicine that, since elderly patients often have multiple and varying comorbidities of varying severity, that measures of function are the most reliable means of predicting outcomes and costs. As such, perhaps more consistent ways to translate functional measures (measures captured in clinical trials fairly reliably) into costs may be the most effective way to conduct cost-effectiveness studies. It is unclear why excess costs were not noted, even in AD patients with at least five comorbidities in the current analyses, since this population is most often frail and has been demonstrated previously to consistently have excess costs as the number of comorbidities rise [9, 12]. This discrepancy suggests that a combination of sources may be most beneficial when evaluating the cost and burden of disease as well as the impact of new treatments on costs of illness. Although the ADCS-ADL is intended to capture functional impairment due to underlying cognitive impairment, it is possible that other comorbidities will impact the rating of the functional scale. Another explanation for the lack of association between comorbidities and cost in the current analysis is that specific comorbidities, which were not collected, may have had stronger associations with cost. Although cost analyses of clinical trial data may not adequately capture the variability in costs due to comorbidity-derived subgroups, clinical trial data may give preliminary indications as to the impact of altering the course of cognitive and functional decline on costs.

The present analyses offer an important step forward in understanding how clinical trials may help inform decisions about the overall impact of new therapeutics beyond traditional regulatory audiences. Notably, there is a paucity of published literature in AD on cost that has been directly garnered from clinical trials. Functional ability remains a consistent predictor of costs in the care of patients with AD. Comorbidities are likely less informative in determining variances in baseline costs if looking at clinical trial populations in isolation. Therefore, it is just as important for trial sponsors to include resource utilization measures in their trials as it is for decision makers to allow extrapolations beyond the clinical trial to best characterize and predict the full risk–benefit profile of a new therapeutic agent. Expanded precision and ease of diagnosis of AD pathology is also likely to move clinical trial populations closer to “typical” patient populations as the current paradigm shifts away from AD as a rule-out diagnosis.
